# Is there adaptation in the ozone mortality relationship: A multi-city case-crossover analysis

**DOI:** 10.1186/1476-069X-7-22

**Published:** 2008-05-30

**Authors:** Antonella Zanobetti, Joel Schwartz

**Affiliations:** 1 Department of Environmental Health, Exposure Epidemiology and Risk Program, Harvard School of Public Health, Boston, MA, USA

## Abstract

**Background:**

Ozone has been associated with daily mortality, mainly in the summer period. Despite the ample literature on adaptation of inflammatory and pulmonary responses to ozone, and the link, in cohort studies, between lung function and mortality risk there has been little done to date to examine the question of adaptation in the acute mortality risk associated with ambient ozone.

**Methods:**

We applied a case-crossover design in 48 US cities to examine the ozone effect by season, by month and by age groups, particularly focusing on whether there was an adaptation effect.

**Results:**

We found that the same day ozone effect was highest in summer with a 0.5% (95% CI: 0.38, 0.62) increase in total mortality for 10 ppb increase in 8-hr ozone, whilst the effect decrease to null in autumn and winter. We found higher effects in the months May- July with a 0.46% (95% CI: 0.24, 0.68) increase in total mortality for 10 ppb increase in ozone in June, and a 0.65% (95% CI: 0.47, 0.82) increase in mortality during July. The effect decreased in August and became null in September. We found similar effects from the age group 51–60 up to age 80 and a lower effect in 80 years and older.

**Conclusion:**

The mortality effects of ozone appear diminished later in the ozone season, reaching the null effect previously reported in winter by September. More work should address this issue and examine the biological mechanism of adaptation.

## Background

While early attention to the acute effects of air pollution on mortality rates focused on particles, previous [1,2] and more recent studies have reported associations with ozone. [3-6] In general, these studies reported associations with ozone, but the associations seemed to be primarily restricted to the summer period [7].

There is ample evidence that short-term ozone exposure is associated with decrements in lung functions, increased respiratory symptoms, and lung inflammation [8]. Some studies showed also that the effect is higher in asthmatic or in individuals with already impaired respiratory function [9-11]. A key finding of these studies was the existence of an adaptive response. For example, several studies found reduced effects of ozone on lung function later in the ozone season [12-15].

Despite this considerable literature on adaptation [12,16,17] of inflammatory and pulmonary responses to ozone, and the link, in cohort studies, between lung function and mortality risk [18-20] there has been little done to date to examine the question of adaptation in the acute mortality risk associated with ambient ozone. In addition, while there have been some publications looking at effects in the elderly Vs less elderly subjects, there has been little to date examining the ozone – mortality association by finer age categories.

In this study we used 48 cities distributed across the U.S. to examine whether there is adaptation to the ozone effect; that is whether repeated or prolonged ozone exposure can induce a decrement in mortality. Specifically, we used a case-crossover design to examine the ozone effect by season, by month and by age groups.

## Methods

We obtained individual mortality data from the National Center for Health Statistics (NCHS) for the years 1989 to 2000, for forty-eight cities in the United States. The mortality files provided information on the exact date of death, and the underlying cause of death; we examined all-cause daily mortality.

We obtained ozone (8-hour mean) data from US Environmental Protection Agency's Air Quality System Technology Transfer Network. Because ozone is often not measured during cold months, we deleted those seasons with less than 75% of days with ozone data in cities where this occurred. We used ozone concentrations on the day of death, which has shown the strongest effects in most previous studies.

We obtained local meteorological data (temperature, and dew point temperature) from the United States Surface Airways and Airways Solar Radiation hourly data [21], and we computed apparent temperature (AT).

To examine the issue of adaptation, we adopted a staged approach. First, we examined the effect of ozone by season, and then by month in the warm season, which is the period where adaptation has been reported for other outcomes, and when previous studies have reported the strongest mortality association with ozone. We also examined effect modification by age categories, again in the May – Sept period.

The case-crossover design compares each subject's exposure experience in a time period just prior to a case-defining event with that subject's exposure at other times. Since there is perfect matching on all measured or unmeasured subject characteristics that do not vary over time there can be no confounding by those characteristics. If in addition, the control days are chosen to be close to the event day, slowly varying subject characteristics are also controlled by matching [22]. We used the time stratified approach proposed by Lumley and Levy [23] in our analysis. We defined the hazard period as the day of death; we chose as control days every third day in the same month and year as the case [7,24]. Since the case day and control day are always in the same month of the same year, this approach facilitates the examination of effect size by month of the year. The data were analyzed using a conditional logistic regression (PROC PHREG in SAS, SAS software release 9.1. 2007, SAS Institute, Cary NC).

In the models we controlled for the same day apparent temperature and indicator variables for day of the week.

To test effect modification by season we included an interaction term between ozone and season defined as winter (December-February), spring (March-May), summer (June-August), autumn (September-November). To examine the effect of ozone by month we included an interaction term between each month and ozone, in an analysis restricted to May – Sept months.

The analysis by age group was done using an interaction with age group, defined in 10 years categories.

In a second stage of the analysis, the city specific results were combined using the meta-regression technique of Berkey and coworkers [25]. The results are expressed as percent increase in deaths for a 10 ppb of 8-hr ozone.

## Results

We examined 48 US cities distributed geographically across the US; the smaller cities in terms of population were Terra Haute, IN, Boulder, CO, Provo/Orem, UT, and Youngstown, OH. The biggest cities were Houston, TX, Chicago, IL, New York City, NY, and Los Angeles, CA. Further information on the choice of cities as well as city-specific descriptive statistics have been previously published [26].

We examined 6,951,395 deaths over the entire year and 2,754,176 deaths over the May-September period. Table 1 presents the descriptive statistics for apparent temperature, ozone and total mortality by season, by month and by age group. Because ozone is not measured through the year, we excluded those cities with less than 75% of observation in each season and therefore we examined 29 cities in winter, 32 in spring, 33 in autumn; all 48 cities had summer measurements.

**Table 1:** Combined descriptive across all cities by season, month and age group

	Mean deaths per day			Apparent temperature			Ozone 8-h		
	
	mean	min	max	mean	min	max	mean	min	max
**Season**									
Winter	36.6	51.5	10.2	4.1	-9.0	19.7	16.5	1.8	40.6
Spring	33.4	50.5	16.9	13.1	-4.4	30.4	41.6	6.1	91.4
Summer	31.1	49.8	15.6	25.7	12.6	35.4	47.8	7.4	103.0
Autumn	32.3	49.8	16.5	15.0	-2.9	31.8	33.5	3.2	91.2
									
**Month**									
May	32.1	17.5	48.0	18.4	6.5	30.4	45.0	9.8	90.2
June	31.5	17.2	48.2	23.9	12.6	33.2	46.9	11.2	94.8
July	31.1	17.3	47.5	26.9	18.2	35.0	48.6	11.9	97.9
August	30.7	16.4	47.3	26.2	17.1	34.0	47.9	9.3	96.0
September	31.1	17.4	47.0	21.7	9.4	31.8	40.0	6.1	90.8
									
**Age group**									
0–20	0.9	0.0	5.8						
21–30	0.4	0.0	3.6						
31–40	1.1	0.1	5.9						
41–50	1.9	0.4	7.7						
51–60	2.9	0.8	9.6						
61–70	5.5	2.2	14.6						
71–80	8.3	4.3	19.1						
> 80	10.3	5.7	22.6						

As expected, there were more deaths in winter, with highest ozone levels in summer, generally peaking in July.

Table 2 shows the results of the case-crossover analyses combined across all cities by season, by month and by age group. We found that the same day ozone effect was null during winter, increased in the spring, and has the highest effect during summer, with a 0.5% (95% CI: 0.38, 0.62) increase in total mortality for 10 ppb increase in same day ozone, while the effect decrease to null in autumn.

**Table 2:** Percent increases for 10 ppb increase in ozone by season, month, and age groups.

	**%**	**95% C.I.**	
**by season**			
Winter	-0.13	-0.56	0.29
Spring	0.35	0.16	0.54
Summer	0.50	0.38	0.62
Autumn	0.05	-0.14	0.24
			
**by month**			
May	0.48	0.28	0.68
June	0.46	0.24	0.68
July	0.65	0.47	0.82
August	0.28	0.11	0.46
September	-0.09	-0.35	0.16
			
**by age group**			
0–20	0.08	-0.42	0.57
21–30	0.10	-0.67	0.87
31–40	0.07	-0.38	0.52
41–50	0.08	-0.27	0.43
51–60	0.54	0.19	0.89
61–70	0.38	0.16	0.61
71–80	0.50	0.32	0.67
> 80	0.29	0.13	0.44

We then concentrated on the warmer months to see how the effect varied by month. We found a 0.48% (95% CI: 0.28, 0.68) increase in total mortality for 10 ppb increase in ozone in May; the effect was similar in June, somewhat increased in July (0.65% (95% CI: 0.47, 0.8) but noticeably decreased in August (0.28% (95% CI: 0.1, 0.5)); in September the effect became null.

We also looked at the effect by age group and we found little evidence of effect up to age 50; we found a 0.54% (95% CI: 0.19, 0.89) increase in total mortality for the age group 51–60. This effect was generally similar up to age 80, with a suggestion of a lower effect in subjects older than 80 years.

## Discussion

In this study we found evidence that the effect of ozone on mortality dropped off later in the ozone season (August and September). This is consistent with the hypothesis that adaptation to the acute changes associated with the mortality risk occurs, although clearly other factors may explain this pattern, which requires further study. As ozone concentrations were higher in August than in June, and only dropped off by 10% in September, this does not appear to be due to lower exposure levels. We also found evidence that the effect of ozone on mortality starts at age 50 and that the magnitude of the risk is relatively stable across most of the age range.

This pattern of change in response suggests that adaptation is not immediate, but takes several months. This is consistent with some literature. For example, one study[27] measured lung function changes and irritant symptoms in LA residents in different seasons and, among other results, found that in fall responders had lost much of their reactivity, as if they had "adapted" to summer ambient O3 exposures. Other studies compared Los Angeles residents response to O3, to Canadians [28] and to new residents [29] and both studies showed that Los Angeles residents had a minimal response, suggesting that exposures to elevated ambient concentrations of O3 produce adaptation in residents of photochemical pollution areas.

Other studies [16,30] showed that even if there is lung function adaptation, lung injury may persist and even cause structural damage to the respiratory tract in humans.

The biological mechanism by which ozone can affect mortality is still under examination. A review of toxicological studies found decreased heart rate, metabolism, blood pressure, and cardiac output when rats are exposed to typical concentrations of ozone [31].

Others studies showed that the respiratory inflammation may inhibit recovery from infection, or produce systemic responses [32-34], supporting a plausible association with cardiovascular mortality.

A study [35] examined whether biomarkers of inflammation are detectable in humans exposed to ozone and associated co-pollutants under natural conditions outdoors by examining 19 normal volunteer joggers with bronchoscopy with bronchoalveolar lavage (BAL). The authors found a possible ongoing inflammatory response in the lungs of recreational joggers exposed to ozone and associated co-pollutants during the summer months.

To the extent that systemic inflammation is driven by inflammation in the lung, and such lung inflammation is involved in stimulation of irritant receptors, and hence the parasympathetic responses, it is possible that the adaptation seen in previous studies of inflammatory changes could result in an attenuation of the mortality risk over the ozone season. Clearly, further work will be required to elucidate this.

Several alternative explanations need to be considered. First, a non-linear dose response relation could appear as an interaction by month if the average concentrations differ by month. Also, heat waves coincide with high ozone levels, and our control for temperature may have been inadequate to capture that. And third, there may be an interaction between ozone and temperature, which could again appear as an interaction with calendar month if the mean temperature differed by month. Regarding the first point, a recent large multi-city study by Bell and coworkers [36] addressed the linearity of the dose-response relation. No significant deviation was found from linearity in multiple models, and a spline model showed a lower slope only at very low ozone concentrations. The ozone concentrations were as high in August as they were in July, and almost as high in September as in May in our study. This, plus the essentially linear relation reported by Bell, makes that explanation for our findings unlikely. To address the second point we performed a sensitivity analysis where we deleted days with temperature over the 99 percentile. The results show that deleting days with high temperature did not change the results.

Finally, we do not think that in our case the different effects found by month was due to an interaction with high temperature. If there was interaction then we would expect a lower effect in May when the mean temperatures are lower, instead we found it in September, when the average temperature is higher than in May.

Then we did perform a meta-analysis dividing the cities among 6 regions and we still found that the effects in May are higher than in September in all regions (results not shown).

By season our analysis produced similar results to previous studies. For example, the meta-analyses of Levy [5] and Ito [6] reported summer effect size estimates of 0.41% and 0.39% for a 10 ppb increase in maximum hourly ozone, compared to our results, with an estimate of 0.5% for a 10 ppb increase in the 8-hour mean ozone during summer. Similarly they also didn't found an ozone effect during colder seasons.

The null effect found in winter and autumn could be explained by the lower levels of ozone in these seasons. Moreover people spend more time indoors; during colder months windows are generally closed, buildings have a lower air exchange rate and in these circumstances the levels of indoor ozone are very low.

One limitation of this study is our inability to control for ambient particles, due to the every sixth day sampling of particles. However, previous studies have reported that PM_10 _is not a confounder [6,7,37].

A recent paper [38] addressed the question of whether the ozone mortality relationship is confounded by secondary particles, which are produced by the same processes that produce ozone and found that the ozone effect didn't change when adjusting for PM_2.5_, OC or nitrate, but did decrease by 25% when adjusting for particle sulfate. Secondary particles are monitored even less than PM10, so we could not directly address the question of whether the differences we see by month could be due to different patterns of correlation by month. However, using the data from the US EPA's Speciation and Trends Network, we examined the correlations between ozone and secondary particles in all reporting cities, by month. We found the correlations between ozone and EC, OC and sulfate were lower in May compared to September. Hence, if anything, there would be more uncontrolled confounding by secondary particles in September than in May, which would be expected to inflate the September coefficient relative to May. Our study found the opposite, and hence secondary particles are unlikely to explain that finding.

This study is the first to look at fine age categories in examining the different age patterns of ozone-associated deaths. We found that the ozone-mortality association began at age fifty, and the 50–59 age group had the highest coefficient. This may be due to chance, as the effect size varied little up until age 80, after which it declined somewhat. This finding is of significance for risk assessment, since years of life lost is greater if the effect begins at age 50 than if it had no impact until after age 65.

## Conclusion

There is considerable literature on adaptation of inflammatory and pulmonary responses to ozone; the association of ozone with daily deaths in the summer is also well established. Not much work has been done to examine the question of adaptation in the acute mortality risk associated with ambient ozone.

The main purpose of the study was to investigate whether there is adaptation to the ozone effect; that is whether repeated or prolonged ozone exposure can induce a decrement in mortality. We found that late in the ozone season, the effect of ozone diminishes, reaching the null effect, previously reported in winter, by September. More work should address this issue and examine the biological mechanism of adaptation.

## Competing interests

The authors declare that they have no competing interests.

## Authors' contributions

AZ participated in the design of the study, prepared the datasets, performed the statistical analysis, and drafted the manuscript.

JS participated in the design of the study, and helped writing the manuscript, revising it critically for important intellectual content.

All authors read and approved the final manuscript.
